# Genetic association and gene expression studies suggest that genetic variants in the *SYNE1* and *TNF* genes are related to menstrual migraine

**DOI:** 10.1186/1129-2377-15-62

**Published:** 2014-10-14

**Authors:** Astrid J Rodriguez-Acevedo, Robert A Smith, Bishakha Roy, Heidi Sutherland, Rod A Lea, Alison Frith, E Anne MacGregor, Lyn R Griffiths

**Affiliations:** 1 Genomics Research Centre, Institute of Health and Biomedical Innovation, Queensland University of Technology, Musk Ave, Kelvin Grove, Brisbane, QLD 4059, Australia; 2 Clinithink Limited, Bridgend, UK; 3 Centre for Neuroscience & Trauma, Blizard Institute of Cell and Molecular Science, London, UK

**Keywords:** Migraine, Menstrual migraine (MM), Menstrual related migraine (MRM), Pure menstrual migraine (PMM), ESR1, PGR, TNF, SYNE1

## Abstract

**Background:**

Menstrual migraine (MM) encompasses pure menstrual migraine (PMM) and menstrually-related migraine (MRM). This study was aimed at investigating genetic variants that are potentially related to MM, specifically undertaking genotyping and mRNA expression analysis of the *ESR1*, *PGR*, *SYNE1* and *TNF* genes in MM cases and non-migraine controls.

**Methods:**

A total of 37 variants distributed across 14 genes were genotyped in 437 DNA samples (282 cases and 155 controls). In addition levels of gene expression were determined in 74 cDNA samples (41 cases and 33 controls). Association and correlation analysis were performed using Plink and RStudio.

**Results:**

SNPs rs3093664 and rs9371601 in *TNF* and *SYNE1* genes respectively, were significantly associated with migraine in the MM population (p = 0.008; p = 0.009 respectively). Analysis of qPCR results found no significant difference in levels of gene expression between cases and controls. However, we found a significant correlation between the expression of *ESR1* and *SYNE1*, *ESR1* and *PGR* and *TNF* and *SYNE1* in samples taken during the follicular phase of the menstrual cycle.

**Conclusions:**

Our results show that SNPs rs9371601 and rs3093664 in the *SYNE1* and *TNF* genes respectively, are associated with MM. The present study also provides strong evidence to support the correlation of *ESR1*, *PGR*, *SYNE1* and *TNF* gene expression in MM.

## Background

Menstrual migraine (MM) encompasses pure menstrual migraine (PMM) and menstrually-related migraine (MRM), which have been recognized as subtypes of migraine without aura (MO) in the International Classification of Headache Disorders (ICHD-III) [1]. The diagnostic criteria are placed in the appendix section of the classification because of uncertainty over whether they should be considered as separate entities. Pure MM is diagnosed when the patient fulfills criteria for MO and has confirmed that attacks occur on day 1 ± 2 of menstruation in at least two out of three menstrual cycles; MRM has the same characteristics as pure MM but attacks occur additionally at other times of the cycle.

Hormonal fluctuations throughout a woman’s life may contribute to the pathophysiology of MM. The main neuroendocrinologic events across the menstrual cycles during the reproductive years involves hypothalamic, pituitary, and ovarian axes. Gonadotropin releasing hormone (GnRH) is synthesized in the hypothalamus and then released into the portal circulation. GnRH binds to receptors in the anterior pituitary gland and activates release of follicle stimulating hormone (FSH) and luteinising hormone (LH). Release of FSH and LH lead to development of follicles within the ovary which produce estrogen during the early to mid-follicular phases (FP) of the menstrual cycle. Serum estrogen levels rise during the late follicular phase to release LH that, in turn, will initiate ovulation within 48 to 72 hours. After ovulation, the remnants of the dominant follicle become the corpus luteum, which then produce moderate to high amounts of both estradiol and progesterone during the luteal phase (LP) of the menstrual cycle. If fertilization and implantation of the ovum do not occur, then the corpus luteum regresses leading to decreasing serum levels of estrogen and progesterone during the late luteal phase causing menstrual bleeding [2]. This drop in estrogen is thought to be an important trigger in MM, first postulated by Somerville in 1972 as the ‘estrogen withdrawal’ theory [3].

The fall in estrogen is believed to be involved in increasing the susceptibility of prime blood vessels to other factors that influence migraineogenic effects through a process that remains unknown [4]. One of these factors may be prostaglandins (PGs), which have been implicated in neurogenic inflammation [5]. PGs may play a role in MM, given that there is a threefold increase in prostaglandin levels by the luteal phase, with a further increase during menstruation [6].

Different genetic variants have been identified as causing migraine. Three genes, *CACNA1A, ATP1A2* and *SCNA1A,* have come from studies performed in individuals with familial hemiplegic migraine (FHM) a monogenic form of migraine with aura [7-9]; *NGF*, *PGCP*, *PRDM16*, *TRPM8*, and *LRP1* are genes recently associated in Genome Wide Association Scan studies (GWAS) in common migraine populations [10]. In addition, *ESR1, ESR2, PGR, AR, FSHR, NRIP1, CYP19A1* and *MTHFR* are genes with variants that have been associated with migraine without aura and these represent special concern for MM researchers due to their role in hormonal processes [11,12]. However, results from these studies have been contradictory and they need to be replicated in different populations. In particular, it is unclear as to the effect of these genes in different migraine subtypes, including MM.

Thus we investigated genetic variants in 14 genes; Follicular stimulating Hormone Receptor (*FSHR*), A CGPR receptor (*RAMP1*), lymphotoxin alpha (*LTA*), Tumor Necrosis Factor (*TNF*), Synaptic Nuclei expressed (*SYNE1*), Potassium Channel Subfamily K Member 18 (*KCNK18*), Estrogen Receptor type 2 (*ESR2*), Cytochrome P450 Family 19, Subfamily A, Polypeptide 1 (*CYP19A1*), Estrogen Receptor 1 (*ESR1*), Progesterone Receptor (*PGR*), Nuclear Receptor Interacting Protein 1 (*NRIP1*) along with microRNA 890 (*MIR890*), microRNA 891A(*MIRF981A*) and microRNA 892A (*MIR892A*) in a population of MM cases and controls. These genes come from neuronal, hormonal and immunologic pathways previously associated with migraine. Genotyping analysis was followed by qPCR for determination of mRNA levels from genes significantly associated with MM in this study.

## Methods

### Population

The population for this research consisted of 437 females recruited by the City of London Migraine Clinic, including both PMM and MRM cases and controls. Migraine diagnosis was in accordance with ICHD-II. The inclusion criteria for cases selection was the occurrence of attacks on day 1 ± 2 of menstruation in at least two out of three menstrual cycles. Diagnosis of PMM (menstrual attacks only) and MRM (additional attacks at other times of the cycle) was confirmed by diary evidence from at least three menstrual cycles. Controls were women with no personal or family history of migraine, age and ethnicity matched to cases, where possible. Biological specimens were collected and transported to the Genomics Research Centre for further processing. Saliva samples were obtained from the 437 females (282 cases: 68 PMM and 214 MRM; and 155 controls (median age 45.0; range 21–60 vs. 39.5; 22–61 years). Saliva samples were collected and DNA isolated using Oragene (Australia) Saliva DNA extraction kits. 74 patients (41 cases and 33 controls (median age 42.0; range 21–49 vs. 35.5; 24–49 years) with paired follicular and luteal phase samples available for 30 cases and 29 controls from the same population, who had not taken any hormonal treatment (including dietary isoflavone supplements) within the previous three months, were asked to donate blood samples at both luteal and follicular menstrual cycle stages. A total number of 134 samples were collected in Paxgene tubes for expression analysis purposes. mRNA extraction was carried out using Qiagen PAXgene blood miRNA kits (Catalog # 763134). Phenotypic data was obtained via a medical questionnaire that surveyed migraine family history, symptoms, triggers, medication use and contraceptive use. The study protocol was originally approved by the Griffith University Human Research Ethics Committee and subsequently by the Queensland University of Technology Human Ethics Committee (Australia), and the East London and the City Research Ethics Committee (UK). All subjects provided signed, informed consent prior to participation.

### Genotype analysis

For this part of the study three different techniques were used. Genotyping of the G594A variant in the *ESR1* gene was carried out by using PCR-RFLP. The restriction enzyme *Btg*I (New England Biolabs, Australia) was used for the determination of the SNP genotype. Variant C325G (rs1801132) was genotyped using TaqMan^®^ SNP Genotyping Assay (life technologies, Cat. #4351379) and following the manufacturer instructions.

PROGINS insertion/deletion was tested with a standard PCR. Samples were then observed on a 2% agarose gel to detect the presence of the PROGINS insert (details in Additional file 1).

A total of 34 SNPs were selected from 14 different genes on nine chromosomes as the research panel for the study. Selected SNPs were located in genes involved in neuronal, hormonal and immunologic pathways previously associated with migraine. The SNPs were tested by using the Sequenom genotyping platform (Sequenom^®^, San Diego, CA, USA), which uses MALDI-TOF mass spectroscopy and MassARRAY technology with an iPlex system. Primers for polymerase chain reaction (PCR) amplification and single base extension were designed by Sequenom Assay Design 3.1 software (Sequenom, San Diego, CA, USA) according to the manufacturer’s instructions (Additional file 2).

### Gene expression analysis

Two-step quantitative reverse transcription PCR (qPCR) was performed using a standardized protocol in our laboratory. An amount of 100 ng of RNA was converted into cDNA by adding 9.2 ng/uL Invitrogen Random Hexamers, dNTPs (500 uM), and free-RNAse H2O to a final volume of 32.5ul. The reaction was incubated at 65°C for 10 minutes in a thermocycler to disrupt secondary structures, followed by a second incubation on ice for at least 5 minutes. In the second step of the reaction, 1st Strand Buffer (1X), DTT (2 mM), SuperScript^®^ III Reverse transcriptase (100U) and RNase- free H2O were added to the previous reaction product for a final volume of 50 ul. The cDNA synthesis reaction was carried out with an initial incubation of 25°C for 5 minutes, followed by a 50°C for 60 minutes and a final step of reaction inactivation at 70°C for 15 min. Stock cDNA was stored at −80°C and 1:2 diluted cDNA working samples were stored at −20°C.

To scan levels of expression of *PGR*, *ESR1*, *SYNE1* and *TNF* genes we used SYBR Green PCR Master Mix 1X (Life Technologies, Australia), 200 nM reverse and forward primers (Additional file 2), Rox (reference probe) 2X, 100 ng of cDNA and free RNase- H2O to a final volume of 10 ul. Fifty cycles of 50°C-2 min, 95°C-3 min, 95°C–3 s, and 60°C–30 s were carried out in a 7900HT Fast Real-Time PCR System (Applied Biosystems, Australia). In order to allow for relative quantitation of gene expression, we used the reference genes 18S and GADPH. Efficiency tests for all primers sets to be used (Additional file 3) were performed and amplification efficiencies were found to be comparable.

### Statistics

As part of the quality control process, we first performed a Hardy Weinberg test followed by a standard case–control association test using Chi-square analysis with Plink V1.07 [13]. In order to minimize the effect of having a dissimilar number of cases and controls in our study, we implemented a logistic regression analysis in RStudio (version 0.97.312) [14] for those SNPs with p-values < 0.01 in the previous Chi-square test. A Wald test was applied to fit the logistic regression model.

For expression analysis the ΔΔCt method was used. The ΔΔCt calculations were executed using Microsoft Excel software. The statistical significance of differentially expressed genes between cases and controls was determined by a standard *t*-test. Logistic regression was also performed using genotype and gene expression levels as predicting variables for migraine. The Pearson’s correlation was used to test correlations between expression levels of *PGR*, *ESR1*, *TNF* and *SYNE1*. The statistical significance was assessed by comparing the observed p values to an alpha threshold of 0.01. All these analyses were performed by using RStudio.

## Results

### Genotyping study

The initial quality control process allowed us to identify and subsequently to delete 6 SNPs for violating the Hardy/Weinberg equilibrium (rs1805087, rs1584243, rs1800683, rs1800629, rs1519480 and rs7127507), 2 SNPs (rs1800630, rs363314) for having a low genotyping calling rate (<80%), 1 SNP with 3 alleles (rs12273363) and 3 SNPs for showing a unique allele (rs146806052, rs113352055 and 5965992). Table 1 shows Chi-square and p-values for the studied SNPs that passed all the quality control checks. The analysis was completed for all cases grouped together (PMM and MRM), and individually for each subgroup. SNPs rs3093664 (*TNF*) and rs9371601 (*SYNE1*) were both significantly associated with migraine in the combined PMM-MRM sample and the MRM sample. Association after exclusion of PMM individuals from the analysis lead to a increased of the level of significance, suggesting a stronger effect of *TNF* and *SYNE1* variants in MRM patients. Significance was detected in two SNPs (rs2229741 and rs4986938), located in NRIP1 and ESR2 in PMM individuals. Table 2 shows allelic and genotypic frequencies and counts for associated SNPs.

**Table 1:** Association analysis

						**All Population**		**MM**		**MRM**	
**Chromosome**	**SNP**	**Gene**	**Alleles**		**HWE**	**CHISQ**	**p-value**	**CHISQ**	**p-value**	**CHISQ**	**p-value**
2	rs6166	FSHR	G	A	0.61	1.94	0.16	2.32	0.13	1.07	0.30
2	rs895572	RAMP1	C	T	0.83	0.88	0.35	0.38	0.54	0.18	0.67
2	rs1080519	RAMP1	T	C	0.66	0.90	0.34	0.06	0.81	0.01	0.92
2	rs10185142	RAMP1	T	C	0.20	0.00	0.96	1.39	0.24	0.23	0.63
2	rs6729271	RAMP1	T	C	0.62	1.99	0.16	0.82	0.37	0.00	0.97
2	rs6707038	RAMP1	G	A	0.84	0.004	0.95	0.01	0.93	0.10	0.75
6	rs2009658	LTA	G	C	0.72	1.75	0.19	2.10	0.15	1.95	0.16
6	rs2071590	LTA	T	C	0.10	0.97	0.32	0.27	0.61	0.98	0.32
6	rs2239704	LTA	T	G	0.26	0.50	0.48	0.01	0.92	0.42	0.52
6	rs909253	LTA	C	T	0.15	1.51	0.22	2.62	0.11	0.95	0.33
6	rs2229094	LTA	C	T	0.10	0.72	0.40	1.45	0.23	0.30	0.58
6	rs3093664	TNF	G	A	1.00	7.00	0.008*	3.26	0.07	7.55	0.006*
6	rs9371601	SYNE1	T	G	0.74	6.65	0.009*	0.12	0.73	9.20	0.002*
6	G594A	ESR1	A	G	0.85	0.62	0.43	0.60	0.44	0.70	0.40
6	C325G	ESR1	G	C	0.89	1.48	0.22	0.07	0.79	1.50	0.22
10	rs140325655	KCNK18	C	T	1.00	0.06	0.81	1.21	0.27	0.67	0.41
10	rs963975	KCNK18	G	C	0.69	3.40	0.07	1.20	0.27	3.15	0.08
11	PROGINS	PgR	D	I	0.25	0.03	0.85	0.48	0.49	0.05	0.82
14	rs4986938	ESR2	G	A	0.41	1.96	0.16	4.82	0.028*	0.83	0.36
15	rs4646	CYP19A1	A	C	0.89	0.55	0.46	0.02	0.89	1.10	0.29
15	rs10046	CYP19A1	C	T	0.31	0.07	0.80	0.06	0.80	0.01	0.92
21	rs2229741	NRIP1	A	G	0.44	2.01	0.16	4.35	0.036*	0.60	0.44
X	rs5965660	MIR890	G	T	0.85	2.83	0.09	0.83	0.36	3.19	0.07
X	rs4827678	MIR890	G	A	0.09	0.34	0.56	0.15	0.70	0.20	0.65
X	rs2202091	MIR890	C	A	0.81	0.44	0.51	1.01	0.31	0.20	0.66

A standard association analysis was performed for all the individuals in the population and for subgroups. PMM (Pure Menstrual Migraine) and MRM (Menstrually-Related Migraine). *bold font indicates significant p-values (p < 0.01).

**Table 2:** Alleles and genotypes frequencies for each of the associated SNPs according to the sub-population MRM, MM or the total population

	**rs3093664**					**rs9371601**				
	**Alleles**		**Genotypes**			**Alleles**		**Genotypes**		
	**G**	**A**	**AA**	**AG**	**GG**	**G**	**T**	**GG**	**GT**	**TT**
Total Population	0.11 (51)	0.89 (493)	0.78 (173)	0.20 (45)	0.01 (3)	0.65 (323)	0.35 (175)	0.41 (102)	0.48 (119)	0.11 (27)
MRM	0.10 (32)	0.90 (268)	0.78 (133)	0.21(35)	0.01(2)	0.66 (233)	0.33 (113)	0.44 (84)	0.47 (91)	0.09 (17)
PMM	0.13(19)	0.87(125)	0.78 (40)	0.20 (10)	0.02 (1)	0.60 (90)	0.40 (62)	0.32 (18)	0.50 (28)	0.18 (10)
Controls	0.20 (25)	0.80 (95)	0.62 (37)	0.35 (21)	0.03 (2)	0.56 (139)	0.44 (113)	0.33 (42)	0.44 (55)	0.23 (29)
	**rs4986938**					**rs2229741**				
	**Alleles**		**Genotypes**			**Alleles**		**Genotypes**		
	**A**	**G**	**AA**	**GA**	**GG**	**A**	**G**	**AA**	**AG**	**GG**
Total Population	0.50 (148)	0.50(148)	0.29 (42)	0.44 (64)	0.28 (41)	0.43 (197)	0.57 (255)	0.20 (44)	0.48 (108)	0.32 (73)
MRM	0.53 (103)	0.47(93)	0.30 (34)	0.46 (51)	0.24 (27)	0.41 (127)	0.59 (181)	0.19 (32)	0.46 (79)	0.35 (61)
PMM	0.46 (46)	0.54 (54)	0.23 (8)	0.37 (13)	0.40 (14)	0.48 (70)	0.52 (74)	0.23 (12)	0.55 (29)	0.23 (12)
Controls	0.58 (75)	0.42 (55)	0.31 (20)	0.54 (35)	0.15 (10)	0.38 (88)	0.62 (144)	0.16 (19)	0.43 (50)	0.41 (47)

Counts are shown in parenthesis next to the frequencies.

Table 3 presents logistic regression analysis results. Both allelic and genotypic odds ratios are shown Genotype GA in SNP rs3093664 showed a protective effect on migraine risk in the total population (OR = 0.46, 95% CI = 0.24-0.86) and in the MRM sub- population (OR = 0.46, 95% CI 0.24-0.89). This effect is likely based on the presence of the G allele, which showed a significant p-value of 0.009 for the odds ratio test in the total population (OR = 0.48, 95% CI = 0.28-0.84) and 0.007 in the MRM sub-population (OR = 0.45, 95% CI = 0.25-0.81), which suggests an important protective effect of this variant on the MRM phenotype. While the model for the GG genotype was not significant and the 95% CI straddled 1, this was likely caused by the extreme rarity of the GG genotype in both case and control populations.

**Table 3:** Odds ratio calculation for all associated SNPs

		**General**			**MRM**			**MM**		
		**OR**	**95% CI**	**p-value**	**OR**	**95% CI**	**p-value**	**OR**	**95% CI**	**p-value**
**rs3093664**	Allele G	0.48	0.28-0.84	0.009	0.45	0.25-0.81	0.007	0.50	0.23-1.07	0.074
	AG vs AA	0.46	0.24-0.86	0.008	0.46	0.24-0.89	0.01	0.44	0.18-1.06	0.03
	GG vs AG	0.7	0.11-4.51	0.99	0.6	0.08-4.58	0.25	1.05	0.08-13	0
	GG vs AA	0.32	0.05-1.99	0.14	0.28	0.04-2.04	0.12	0.46	0.04-5.32	0.51
**rs9371601**	Allele T	0.48	0.28-0.84	0.009	0.602	0.43-0.84	0.003	0.93	0.60-1.43	0.73
	GT vs GG	0.89	0.55-1.44	0.76	0.83	0.5-1.36	0.4	1.19	0.58-2.43	0.76
	TT vs GT	0.43	0.23-0.8	0.003	0.35	0.18-0.7	0.001	0.68	0.29-1.59	0.29
	TT vs GG	0.38	0.2-0.72	0.001	0.29	0.14-0.59	p < 0.001	0.8	0.33-1.99	0.76
**rs4986938**	Allele G	1.32	0.88-1.99	0.17	1.22	0.78-1.90	0.37	1.90	1.05-3.47	0.03
	GA vs AA	0.87	0.44-1.71	0.92	0.86	0.43-1.73	0.83	0.93	0.33-2.62	2.79
	GG vs GA	2.24	1-5.01	0.02	1.85	0.8-4.31	0.09	3.77	1.34-10.57	0.005
	GG vs AA	1.95	0.82-4.67	0.08	1.59	0.64-3.95	0.24	3.5	1.1-11.09	0.017
**rs2229741**	Allele A	1.25	0.911-1.72	0.16	1.13	0.811-1.59	0.45	1,616	1.01-2.56	0.04
	AG vs GG	1.39	0.85-2.29	0.13	1.22	0.72-2.05	0.4	2.27	1.04-4.96	0.02
	AA vs AG	1.07	0.57-2.02	1.76	1.07	0.55-2.08	1.95	1.09	0.46-2.56	0.04
	AA vs GG	1.49	0.78-2.86	0.16	1.3	0.66-2.57	0.4	2.47	0.95-6.47	0.03

The analysis was carried out in the total population as well as in MRM and PMM subpopulations.

Additionally, allele T in SNP rs9371601 showed a significant OR in MRM (OR = 0.60, 95% CI = 0.43-0.84) and not the PMM sub-population (OR = 0.93, 95% CI = 0.60-1.43) (see Table 2), indicating that the effects of this SNP may be largely confined to the MRM sub-population. Interestingly, the genotypic odds ratio indicated that this protective effect was limited to TT genotypes and that heterozygotes did not show confident protection compared to GG homozygotes (OR = 0.83, 95% CI = 0.50-1.36). Allele G in SNP rs4986938 (OR = 1.90, 95% CI = 1.05-3.47) and allele A in SNP rs2229741 (OR = 1.61, 95% CI = 1.01-2.56) seemed to be a risk factor for migraine in the PMM sub-population. However, the effect is probably not strong due to the small size of the population.

### Expression study

Our analysis of gene expression in our migraine populations indicated no significant difference in levels of expression in the studied genes between cases and controls and across the sub-populations (Table 4). However, we have detected significant positive correlations in the follicular phase between the expression of *TNF* and *SYNE1* (Rho = 0.371, p = 0.005), *ESR1* and *PGR* (Rho = 0.435, p = 0.006) and *ESR1* and *SYNE1* (Rho = 0.412, p = 0.002), which would indicate some interaction between them. In contrast, in luteal phase, we found a correlation between *SYNE1* and *PGR* (Rho = 0.444, p = 0.006) and *TNF* and *SYNE1* (Rho = 0.345, p = 0. 015). More interestingly, we found differences in the correlations between gene expression in cases and controls. For *ESR1* and *SYNE1*, these differences were small, with both cases and controls maintaining similar levels of correlation (cases: Rho = 0.48 p < 0,001; controls: Rho = 0.4 p = 0.001). For other gene correlations however, cases maintained significant relationships while the controls had weakened or non-significant relationships. For *TNF* and *SYNE1*, the relationship was weaker, but still significant in controls (cases: Rho = 0.449 p < 0,001; controls: Rho = 0.27 p = 0.03). For *ESR1* and *PGR* controls ceased to maintain correlation of expression (cases: Rho = 0.511 p < 0,001; controls: Rho = 0.091 p = 0.56) and a similar loss of correlation occurred between *PGR* and *SYNE1* (cases: Rho = 0.393 p = 0.005; controls: Rho = 0.243 p = 0.12) (Table 5).

**Table 4:** T-student analysis for means difference in levels of gene expression

**Group 1 vs group 2**	**Transcript**	**Mean group 1**	**Mean group 2**	**Difference**	**t.stat**	**t.pval**
**Cases vs Controls**	***TNF***	16.77	17.14	-0.37	-1.12	0.27
	***SYNE1***	20.90	20.36	0.54	0.84	0.41
	***ESR1***	16.35	16.16	0.19	0.29	0.78
	***PGR***	9.61	9.23	0.39	0.33	0.74
**CasesF vs CasesL**	***TNF***	17.16	16.24	0.92	1.53	0.14
	***SYNE1***	21.56	20.01	1.55	1.20	0.24
	***ESR1***	15.98	16.59	-0.60	-0.59	0.56
	***PGR***	9.62	8.95	0.67	0.40	0.69
**CasesF vs ControlsF**	***TNF***	17.16	17.33	-0.17	-0.51	0.61
	***SYNE1***	21.56	20.68	0.89	1.18	0.24
	***ESR1***	15.98	16.73	-0.75	-0.96	0.34
	***PGR***	9.62	9.80	-0.18	-0.11	0.91
**CasesL vs ControlsL**	***TNF***	16.24	16.96	-0.72	-1.14	0.26
	***SYNE1***	20.01	20.06	-0.05	-0.04	0.97
	***ESR1***	16.59	15.62	0.96	0.84	0.40
	***PGR***	8.95	8.69	0.26	0.15	0.88

All the subpopulations were compared. TP: Total Population; F: Follicular phase; L: Luteal Phase.

**Table 5:** P-values after correlating the genes in the middle of the graphic

	**Luteal phase**					**Controls**			
**Follicular Phase**	**ESR1**	0,0105*	0,081	0,605	**Cases**	**ESR1**	0.0039*	0.0014*	0.567
	0,0761	**TNF**	0,0271*	0,315		0.039*	**TNF**	0.0307*	0.5159
	0,0019*	0,0053*	**SYNE1**	0,0058*		0.0003*	0.00012*	**SYNE1**	0.1157
	0,0064*	0,84	0,1039	**PgR**		0.00017*	0.44	0.0047*	**PgR**

Each value represent the correlation significance between two genes. From column 2-5 p-values for correlation between genes in follicular phase and in luteal phase are shown; columns 7-10 show p-values for correlation between cases and controls. Genes are indicated in bold font. * indicates p < 0.05.

The results of our expression analysis suggest that although there are not significant changes in gene expression of genes that may influence migraine in PMM and MRM cases compared to controls, these genes interact in a different fashion both in the luteal and follicular stages of the menstrual cycle and in cases compared to controls.

## Discussion

There have been very few studies carried out so far at the genomic level on MM, in part because of the uncertainty concerning its status as an entity independent from common migraine. In this research, we have identified significant differences between MM cases and controls, which both show increased risk of migraine development for the G alleles in the tested SNPs in the *TNF* and *SYNE1* genes. *TNF* is a pro- inflammatory cytokine and is thus involved in a number of biological pathways, including apoptosis, chemotaxis and cell proliferation. The gene has been associated with neuronal damage and pain in response to particular cellular states, such as hypothermia and hypoxia, and thus has a significant linkage to metabolic pathways that may influence menstrual migraine development and migraine initiation [15]. This is particularly so in view of the influence of estrogen and progesterone on inflammatory processes [16,17], and this association may represent a link to a direct mechanism by which MM is triggered. In addition, *TNF* has been implicated in depression and irritable bowel disease [18,19], recognized as migraine comorbidities [20,21]. The rs3093664 SNP itself is an intronic polymorphism located between exons 3 and 4 of *TNF* away from intron splicing sites, so it is unlikely that the SNPitf has a directeffectt on *TNF* function that would explain the association we have observed. It is thus likely that this result is caused by linkage to a nearby rare SNP that does have functional effects. Interestingly, there is a candidate for this only 52 base pairs upstream of rs3093664, in the form of rs1800620, which is located in exon 3 of *TNF* and causes an alanine to threonine transition in amino acid 94 of the protein. Despite being annotated as a SNP, however, Hapmap populations show no variation in rs1800620 in tested African, Caucasian, Hispanic or Pacific Rim populations and nor do any publications mention it, so its status, and any linkage to rs3093664 remains uncertain.

*SYNE1* is a spectrin repeat containing protein usually found on the nuclear membrane and is involved in several kinds of protein-protein interactions where it serves as a scaffold and chaperone for various binding partners. The gene has been associated with several neural diseases, including depression and cerebellar/spinocerebellar ataxias, which have overlap with the familial forms of migraine driven by mutations, and may have direct effects in menstrual migraine development through these functions [22]. Interestingly, *SYNE1* is also directly adjacent to the estrogen receptor, and polymorphisms within *SYNE1* have been linked strongly to estrogen mediated events, such as ovarian cancer [23]. It is thus possible that the association seen with MM here may represent linkage to estrogen related effects, either through cross-regulation effects of the two genes or via linkage to another marker in the distal parts of *ESR1* that does not show linkage to those markers we have already interrogated in this study. This hypothesis is further supported by the fact that rs9371601 is also an intronic polymorphism, lying between exons 13 and 14 of the gene. Like our tested *TNF* polymorphism, rs9371601 also has potential SNPs it may be linked to which cause amino acid changes with undetermined frequencies, though these are more distant, the closest two being at least 2 kb away up and downstream of it (rs267600867 and rs139324183).

Significant associations were also detected in two SNPs (rs2229741 and rs4986938) in MM individuals, although their significance was not less than 0.01 and due to our relatively small sample sub population for MM samples, we may have impaired capability to detect true associations for this population. We do however; believe it is worthy to further study these SNPs in larger populations, as the genes containing these variants (*NRIP1* and *ESR2* respectively) are important hormonal receptors and modulators of hormonal action that might represent interesting targets in the etiology of menstrual migraine. Like our other 2 SNPs, rs2229741 and rs4986938 are not amino acid changing variants, being intronic and part of the 3′ UTR of their genes respectively, and it is possible that the weakness of the associations we have identified also indicate linkage to functional polymorphisms nearby.

The *ESR1* and *PGR* markers tested in MM showed no significant association in the present study, despite previous association with migraine without aura [24-26]. It is possible that while the functional changes brought about by the tested markers affect hormonal signalling, they do not result in the kind of metabolic changes triggering menstrually related migraine. This is further supported by the different associations we obtained in the PMM and MRM sub-populations. The SNPs in *NRIP1* and *ESR2* are far from significant in the MRM sub populations and while the *SYNE1* SNP is highly non- significant in the MM sub-population, the *TNF* SNP shows near significance in the PMM cases (p = 0.07), indicating a potential, if weaker, effect for this gene in that sub- population. Our results may thus indicate that migraine without aura and MRM may have different causative genes, despite sharing alterations to the hormone receptor pathways as part of their etiology [27].

Statistical analysis showed that there was no significant difference in *ESR1*, *PGR*, *SYNE1* or *TNF* expression for cases or controls in any menstrual phase. We have, however, shown an interesting correlation of expression of the four proteins. Our analysis showed that expression of *ESR1* correlates significantly with *PGR* and *SYNE1*, and also *SYNE1* and *NF* are significantly correlated in the follicular phase of the menstrual cycle. However, this correlation breaks down in the luteal phase where *ESR1* correlates with *TNF* and *SYNE1* correlates with *PGR*. This may represent normal expression control responses for each of the particular genes in response to the altering hormonal situation in the different phases. On the other hand, after comparing expression correlation in cases and controls separately, a change in the complex network of protein interaction between *ESR1*, *TNF*, *PGR* and *SYNE1* seems to be occurring. Highly significant p-values and stronger correlation for *TNF*/*SYNE1*, *ESR1*/*PGR* and *PGR*/*SYNE1* in cases suggest an important role of pathways involving these genes in MM. Maintaining this correlation indicates that individuals suffering from MRM may have increased sensitivity to variations in hormonal signaling effects compared to controls. This is the first time these correlations and their relationship to migraine status has been identified.

Since *SYNE1* polymorphisms have been previously associated with estrogen related events, an expression link between the pro-inflammatory cytokine *TNF* with *SYNE1* in cases might explain why polymorphisms in both genes are associated with MM in our population. This adds some weight to the potential for this pathway to be playing a role in MM and may represent the differential effects of the polymorphisms examined or more likely linked SNPs nearby, on pathway signal transduction. Identifying the specific interactions between *ESR1, PGR, TNF* and *SYNE1* proteins and how polymorphisms factor into them would help to elucidate important mechanisms involved in MM that may be a useful avenue for development of a treatment for the disorder. Further work should also examine additional time points within the follicular and luteal phases to determine if a specific point of departure between case and control gene regulation can be identified.

## Conclusions

Our results show that SNPs rs9371601 and rs3093664 in the *SYNE1* and *TNF* genes, respectively are associated with MRM in our population. Significant associations were also detected in SNPs rs2229741 and rs4986938 in PMM individuals, but due to the small number of PMM samples used to implement the analysis, we should be cautious about interpretation and consider both polymorphisms for future analysis in bigger populations. Statistical analysis showed that there was no significant difference between *ESR1*, *PGR*, *SYNE1* or *TNF* expression in cases or controls in any of the menstrual phases. We have, however, shown a correlation of the four expressed genes in patients with PMM or MRM. Further studies should be focused on the validation of these results in larger populations with more collection timepoints for blood samples and on the understanding of protein interaction in different stages of the menstrual cycle and its effect on migraine etiology. Although we did not find any association of *ESR1* and *PGR* variants with migraine, we believe that future studies should continue exploring the large family of co-activators and co-repressors that modulate the effects of *ESR1* and *PGR*. It may be that variants in these genes result in the development of MRM. Our results also indicate that migraine without aura and MRM may have different causative genes, despite both potentially sharing alterations in hormone receptor pathways [27].

## Abbreviations

FHM: Familial hemiplegic migraine; MO: Migraine without aura; MRM: Menstrually- related migraine; PMM: Pure menstrual migraine; MM: Menstrual migraine.

## Competing interests

The City of London Migraine Clinic received funding from Merck for clinical trials with telcagepant in migraine.

## Authors’ contributions

AF and AM G were responsible for sample collection. AF, AM and LG conceived and supervised the project and edited the manuscript. RS, AR, LG drafted the manuscript. AR, RS, RL performed the statistical analyses. AR, BR and HS carried out the molecular genetic studies. All authors read and approved the final manuscript.

## Supplementary Material

Additional file 1 Corresponding protocol for G594A and PROGINS variants genotyping. Click here for file

Additional file 2 Primer sequences design with Sequenom Assay Design 3.1 software (Sequenom, San Diego, CA, USA). Click here for file

Additional file 3 Primer sequences for qPCR assay. Click here for file

## References

[B1] Headache Classification Committee of the International Headache S The international classification of headache disorders, 3rd edition (beta version) Cephalalgia 2013 33 9 629 808 doi:10.1177/0333102413485658 23771276 10.1177/0333102413485658

[B2] Martin VT Behbehani M Ovarian hormones and migraine headache: understanding mechanisms and pathogenesis–part I Headache 2006 46 1 3 23 doi:10.1111/j.1526-4610.2006.00309.x 10.1111/j.1526-4610.2006.00309.x 16412147

[B3] Somerville BW The influence of progesterone and estradiol upon migraine Headache 1972 12 3 93 102 10.1111/j.1526-4610.1972.hed1203093.x 5075463

[B4] MacGregor EA Frith A Ellis J Aspinall L Hackshaw A Incidence of migraine relative to menstrual cycle phases of rising and falling estrogen Neurology 2006 67 12 2154 8 PubMed PMID: 16971700 10.1212/01.wnl.0000233888.18228.19 16971700

[B5] Antonova M Wienecke T Olesen J Ashina M Prostaglandins in migraine: update Curr Opin Neurol 2013 26 3 269 275 doi:10.1097/WCO.0b013e328360864b 10.1097/WCO.0b013e328360864b 23519238

[B6] Downie J Poyser NL Wunderlich M Levels of prostaglandins in human endometrium during the normal menstrual cycle J Physiol 1974 236 2 465 472 16992446 10.1113/jphysiol.1974.sp010446 PMC1350813

[B7] Ambrosini A D’Onofrio M Grieco GS Di Mambro A Montagna G Fortini D Nicoletti F Nappi G Sances G Schoenen J Buzzi MG Santorelli FM Pierelli F Familial basilar migraine associated with a new mutation in the ATP1A2 gene Neurology 2005 65 11 1826 1828 doi:10.1212/01.wnl.0000187072.71931.c0 10.1212/01.wnl.0000187072.71931.c0 16344534

[B8] Dichgans M Freilinger T Eckstein G Babini E Lorenz-Depiereux B Biskup S Ferrari MD Herzog J van den Maagdenberg AM Pusch M Strom TM Mutation in the neuronal voltage-gated sodium channel SCN1A in familial hemiplegic migraine Lancet 2005 366 9483 371 377 doi:10.1016/S0140-6736(05)66786-4 10.1016/S0140-6736(05)66786-4 16054936

[B9] Ophoff RA Terwindt GM Vergouwe MN van Eijk R Oefner PJ Hoffman SM Lamerdin JE Mohrenweiser HW Bulman DE Ferrari M Haan J Lindhout D van Ommen GJ Hofker MH Ferrari MD Frants RR Familial hemiplegic migraine and episodic ataxia type-2 are caused by mutations in the Ca2+ channel gene CACNL1A4 Cell 1996 87 3 543 552 10.1016/S0092-8674(00)81373-2 8898206

[B10] Silberstein SD Dodick DW Migraine genetics–a review: part I Headache 2013 53 8 1207 1217 doi:10.1111/head.12156 10.1111/head.12156 23848970

[B11] Russell MB Genetics of menstrual migraine: the epidemiological evidence Curr Pain Headache Rep 2010 14 5 385 388 doi:10.1007/s11916-010-0142-6 10.1007/s11916-010-0142-6 20697844 PMC2939351

[B12] Colson N Fernandez F Griffiths L Genetics of menstrual migraine: the molecular evidence Curr Pain Headache Rep 2010 14 5 389 395 doi:10.1007/s11916-010-0129-3 10.1007/s11916-010-0129-3 20625856

[B13] Purcell S Neale B Todd-Brown K Thomas L Ferreira MA Bender D Maller J Sklar P de Bakker PI Daly MJ Sham PC PLINK: a tool set for whole-genome association and population-based linkage analyses Am J Hum Genet 2007 81 3 559 575 doi:10.1086/519795 10.1086/519795 17701901 PMC1950838

[B14] Team RC R: A Language and Enviroment for Statistical Computing 2012 Austria, Vienna

[B15] Christopherson K 2nd Hromas R Chemokine regulation of normal and pathologic immune responses Stem Cells 2001 19 5 388 396 doi:10.1634/stemcells.19-5-388 10.1634/stemcells.19-5-388 11553847

[B16] Hughes GC Progesterone and autoimmune disease Autoimmun Rev 2012 11 6–7 A502 514 doi:10.1016/j.autrev.2011.12.003 22193289 10.1016/j.autrev.2011.12.003 PMC3431799

[B17] Oertelt-Prigione S Immunology and the menstrual cycle Autoimmun Rev 2012 11 6–7 A486 492 doi:10.1016/j.autrev.2011.11.023 22155200 10.1016/j.autrev.2011.11.023

[B18] Dowlati Y Herrmann N Swardfager W Liu H Sham L Reim EK Lanctot KL A meta-analysis of cytokines in major depression Biol Psychiatry 2010 67 5 446 457 doi:10.1016/j.biopsych.2009.09.033 10.1016/j.biopsych.2009.09.033 20015486

[B19] Bashashati M Rezaei N Bashashati H Shafieyoun A Daryani NE Sharkey KA Storr M Cytokine gene polymorphisms are associated with irritable bowel syndrome: a systematic review and meta-analysis Neurogastroenterol Motil 2012 24 12 1102 e1566 doi:10.1111/j.1365-2982.2012.01990.x 10.1111/j.1365-2982.2012.01990.x 22897390

[B20] Cole JA Rothman KJ Cabral HJ Zhang Y Farraye FA Migraine, fibromyalgia, and depression among people with IBS: a prevalence study BMC Gastroenterol 2006 6 26 doi:10.1186/1471-230X-6-26 10.1186/1471-230X-6-26 17007634 PMC1592499

[B21] Victor TW Hu X Campbell J White RE Buse DC Lipton RB Association between migraine, anxiety and depression Cephalalgia 2010 30 5 567 575 doi:10.1111/j.1468-2982.2009.01944.x 19614684 10.1111/j.1468-2982.2009.01944.x

[B22] Noreau A Bourassa CV Szuto A Levert A Dobrzeniecka S Gauthier J Forlani S Durr A Anheim M Stevanin G Brice A Bouchard JP Dion PA Dupre N Rouleau GA SYNE1 mutations in autosomal recessive cerebellar ataxia JAMA 2013 doi:10.1001/jamaneurol.2013.3268 10.1001/jamaneurol.2013.3268 23959263

[B23] Doherty JA Rossing MA Cushing-Haugen KL Chen C Van Den Berg DJ Wu AH Pike MC Ness RB Moysich K Chenevix-Trench G Beesley J Webb PM Chang- Claude J Wang-Gohrke S Goodman MT Lurie G Thompson PJ Carney ME Hogdall E Kjaer SK Hogdall C Goode EL Cunningham JM Fridley BL Vierkant RA Berchuck A Moorman PG Schildkraut JM Palmieri RT Cramer DW Ovarian cancer association C (2010) ESR1/SYNE1 polymorphism and invasive epithelial ovarian cancer risk: an ovarian cancer association consortium study Cancer Epidemiol Biomarkers Prev 19 1 245 250 doi:10.1158/1055-9965.EPI-09-0729 20056644 10.1158/1055-9965.EPI-09-0729 PMC2863004

[B24] Colson NJ Lea RA Quinlan S MacMillan J Griffiths LR Investigation of hormone receptor genes in migraine Neurogenet 2005 6 1 17 23 doi:10.1007/s10048-004-0205-0 10.1007/s10048-004-0205-0 15654614

[B25] Colson NJ Lea RA Quinlan S MacMillan J Griffiths LR The estrogen receptor 1 G594A polymorphism is associated with migraine susceptibility in two independent case/control groups Neurogenet 2004 5 2 129 133 doi:10.1007/s10048-004-0181-4 10.1007/s10048-004-0181-4 15133719

[B26] Gervil M Ulrich V Kaprio J Olesen J Russell MB The relative role of genetic and environmental factors in migraine without aura Neurology 1999 53 5 995 999 10.1212/WNL.53.5.995 10496258

[B27] Mathew PG Dun EC Luo JJ A cyclic pain: the pathophysiology and treatment of menstrual migraine Obstet Gynecol Surv 2013 68 2 130 140 doi:10.1097/OGX.0b013e31827f2496 10.1097/OGX.0b013e31827f2496 23417219

