# Supplementary material for: Genetic association and gene expression studies suggest that genetic variants in the SYNE1 and TNF genes are related to menstrual migraine
Source: J Headache Pain. 2014 Oct 14;15(1):62. doi: 10.1186/1129-2377-15-62 (PMC4196204; doi:10.1186/1129-2377-15-62)
Supplement: Additional file 1 — Corresponding protocol for G594A and PROGINS variants genotyping. [file 1129-2377-15-62-S1.docx]

**Additional file 1. Corresponding protocol for G594A and PROGINS variants genotyping.**

Methodology

Genotype analysis

G594A

Genotyping of the G594A variant in ESR1 gene was carried out by using PCR-RFLP. DNA samples (20ng/uL) were incubated in a 20 uL reaction with dNTPs (0.2mM), 5X buffer, MgCl2 (3.75mM), reverse primer (5’GCC ATT GGT GTT GGA TGC ATG C3’) and forward primer (5’GAG GAG ACG GAC CAA AGC CAC3’) (0.25uM), 5U of Taq Polymerase and water under the following cycling conditions: an initial step at 94°C for 2 min followed by 5 cycles of 94°C for 45 s, 69°C for 1 min, and 72°C for 2 min; then 30 cycles of 94°C for 30 s, 67°C for 30 s and 72°C for 45 s and a final step of 72°C for 5 min. The restriction enzyme BtgI (New England Biolabs, Australia) was used for the determination of the SNP genotype.

PROGINS

PROGINS insertion/deletion was tested with a standard PCR. DNA samples (20ng/uL) were incubated in a 25 uL reaction with dNTPs (0.2mM), 5X buffer, MgCl2 (1.4 mM), reverse primer -5'-AAA GTA TTT TCT TGC TAA ATG TC-3'- and forward primer -5'-GGC AGA AAG CAA AAT AAA AAG A-3'-(0.24uM), 6U of Taq Polymerase (Invitrogen) and water under the following cycling conditions: an initial step at 94°C for 4 min followed by 30 cycles of 94°C for 30 s, 51°C for 30 s, and 72°C for 45 s and a final step of 72°C for 2 min. Samples were then observed on a 2% agarose gel to detect the presence of the PROGINS insert.
