# Supplementary material for: Genetic association and gene expression studies suggest that genetic variants in the SYNE1 and TNF genes are related to menstrual migraine
Source: J Headache Pain. 2014 Oct 14;15(1):62. doi: 10.1186/1129-2377-15-62 (PMC4196204; doi:10.1186/1129-2377-15-62)
Supplement: Additional file 2 — Primer sequences design with Sequenom Assay Design 3.1 software (Sequenom, San Diego, CA, USA). [file 1129-2377-15-62-S2.docx]

**Additional file 2. Primer sequences design with Sequenom Assay Design 3.1 software (Sequenom, San Diego , CA, USA).**

| **SNP** | **chr** | **Forward Primer Sequence** | **Reverse Primer Sequence** | **Extended Primer Sequence** |
| --- | --- | --- | --- | --- |
| rs1805087 | 1 | ACGTTGGATGCTTTGAGGAAATCATGGAAG | ACGTTGGATGTACCACTTACCTTGAGAGAC | aatgACCTTGAGAGACTCATAATGG |
| rs6166 | 2 | ACGTTGGATGAAGGAATGGCCACTGCTCTT | ACGTTGGATGGGGCTAAATGACTTAGAGGG | CAGCTCCCAGAGTCACCA |
| rs895572 | 2 | ACGTTGGATGTTTCACTGTTTTTGGAGCCC | ACGTTGGATGGTCAAAGAAAGTTCTTCAGTG | aacAGTTCTTCAGTGATAAGAAAGA |
| rs1584243 | 2 | ACGTTGGATGGTCTTTTTTCCTCTGACCTC | ACGTTGGATGGACACATCAAACTCCAACTG | ggggTCAAACTCCAACTGCTGAGCACC |
| rs1080519 | 2 | ACGTTGGATGCTCTTTGTGTTCCAGTTTG | ACGTTGGATGCCATCAGCAGACACGAAAAC | gggcAACTGAGGAAAGAGAGAAATT |
| rs10185142 | 2 | ACGTTGGATGGGAGCTTCTCACACGATTTC | ACGTTGGATGGCTGCAGGTTGTCTGAGAG | TTTCGACCTGCGATAC |
| rs6729271 | 2 | ACGTTGGATGCCATGTCATAAAATGTCAC | ACGTTGGATGCCCTTGCTTTTTAAAGCTTTT | ATGTCATAAAATGTCACAGAAATC |
| rs6707038 | 2 | ACGTTGGATGCTCGGTCTCAAAAAACAAAGC | ACGTTGGATGGAATGGTTGGAATTAAGACGG | AAGACGGTTTGTTTACCTG |
| rs2009658 | 6 | ACGTTGGATGAGCTCCAACCCCTCTAACAC | ACGTTGGATGGTCACCTCAAATATTATTAC | ccCCTCAAATATTATTACTGCTACT |
| rs2071590 | 6 | ACGTTGGATGAAGGGACAGTCAATTCAGAG | ACGTTGGATGATGATTGCTCTTCAGGGAAC | ggccGTCAATTCAGAGAGGAGG |
| rs1800683 | 6 | ACGTTGGATGTCTATAAAGGGACCTGAGCG | ACGTTGGATGACGGGCAGCCCAAGGAGAT | gaatGAGAGCCTCACCTGCTGTG |
| rs2239704 | 6 | ACGTTGGATGCAGCAGGTGCAGGAGGGAC | ACGTTGGATGGTGCTTCGTGCTTTGGACTA | TTTGGACTACCGCCC |
| rs909253 | 6 | ACGTTGGATGAGAGACAGGAAGGGAACAGA | ACGTTGGATGTCCATCTGTCAGTCTCATTG | cGAAGGGAACAGAGAGGAA |
| rs2229094 | 6 | ACGTTGGATGTGACACCACCTGAACGTCTC | ACGTTGGATGAGAAGGAGGAGGTGTAGGGT | cccttAACGTCTCTTCCTCCCAAGGGTG |
| rs1800630 | 6 | ACGTTGGATGGCAATGGGTAGGAGAATGTC | ACGTTGGATGCCTCTACATGGCCCTGTCTT | ggggtAAGTCGAGTATGGGGACCCCC |
| rs1800629 | 6 | ACGTTGGATGGGAGGCAATAGGTTTTGAGG | ACGTTGGATGTTCTGGGCCACTGACTGATT | gagtACCCTGGAGGCTGAACCCCGTCC |
| rs3093664 | 6 | ACGTTGGATGAAGAGCTCTGAGGATGTGTC | ACGTTGGATGAAGTTCTGCCTACCATCAGC | tGGCTAGGATTTGGGG |
| rs9371601 | 6 | ACGTTGGATGACACATGGGTGCTCAGTAG | ACGTTGGATGATGAAGTGCAGATACCAGCC | ctttCAGATACCAGCCACTAGAA |
| rs140325655 | 10 | ACGTTGGATGGGTTTAACAGGACCACACAC | ACGTTGGATGACTTACCCACGGTGCTGAAC | TGAGCTCGCTCTTTTTC |
| rs963975 | 10 | ACGTTGGATGCTGTGACTATCCCATTCCAC | ACGTTGGATGTTTGAGGTTTGACCTCAGCC | ggaaTCCCTCACAGTGATTCC |
| rs363314 | 10 | ACGTTGGATGCGCATCCCTTCCTTTGCTTG | ACGTTGGATGTGTCACCCTCAGGAGCATC | ttgaTTGGCTGCCTGGTTGTC |
| rs1519480 | 11 | ACGTTGGATGCTGAAGAGTAAGAACAGATGC | ACGTTGGATGCTTAGGGAAATAAATGGAAGG | TTTTTTCCTTAATGGCCC |
| rs7127507 | 11 | ACGTTGGATGTTAAAACATTCAAGCTTCC | ACGTTGGATGGAGAGAATAGAGAGTTGCGG | TTCAAGCTTCCTTTCTACAA |
| rs12273363 | 11 | ACGTTGGATGGCTATTGACTGCAGGGATGA | ACGTTGGATGGCTGGGTGGTCTGAAACTTA | CGATGCTGCAGAAGA |
| rs4986938 | 14 | ACGTTGGATGAGGTGAACTGGCCCACAGAG | ACGTTGGATGACAGCAGAAAGATGAAGCCC | tggtAGTTCACGCTTCAGC |
| rs4646 | 15 | ACGTTGGATGTCTCTTGTAGCCTGGTTCTC | ACGTTGGATGTACCTCCTATGGGTTGTCAC | tcgCTGGTGTGAACAGGAGCAGATGAC |
| rs10046 | 15 | ACGTTGGATGTCTGGAACACTAGAGAAGGC | ACGTTGGATGGGATGGATGATTTGTATGTG | GAGAAATGCTCCAGAGT |
| rs2229741 | 21 | ACGTTGGATGACCTGTCAAAGTAATGGTCC | ACGTTGGATGGTCTGGCTTTTTTGAGGTGC | gaggTCTCAATACACATACATATCAGGG |
| rs5965660 | X | ACGTTGGATGTCTGAGGACCACCACAAAAG | ACGTTGGATGGATTACATGTGACTGACACC | ggggACCACAAAAGTGTACTTGG |
| rs4827678 | X | ACGTTGGATGTCTTCTGGCATTCGAGAAGG | ACGTTGGATGAACTTTTGGCTGGCCTTTGG | ggggaTCCTGATGCAGTGGAG |
| rs146806052 | X | ACGTTGGATGATATACATGGTGAGCCTTGC | ACGTTGGATGCTACTCAGAAAGGTGCCAT | tgaggGGTGAGCCTTGCTCTACCC |
| rs2202091 | X | ACGTTGGATGGGAAGAGTCACACATCCCTA | ACGTTGGATGGGCCTATCTTGGGTTTTGAC | CTCACTAATCTTAATCAGTTTTTAAT |
| rs113352055 | X | ACGTTGGATGAGTAATACAGTCTCTACCGC | ACGTTGGATGTCTCCTTCACCTCCTTGTAG | AGTGACACTGGTAGTAGA |
| rs5965992 | X | ACGTTGGATGTTTGTAGACTCTTTGGGAAC | ACGTTGGATGGGTATTCAATGTTGCATAGTC | TAATTACGTAGCTTCTTTGTTT |
