# Supplementary material for: Genetic association and gene expression studies suggest that genetic variants in the SYNE1 and TNF genes are related to menstrual migraine
Source: J Headache Pain. 2014 Oct 14;15(1):62. doi: 10.1186/1129-2377-15-62 (PMC4196204; doi:10.1186/1129-2377-15-62)
Supplement: Additional file 3 — Primer sequences for qPCR assay. [file 1129-2377-15-62-S3.docx]

| **Gene** | **Forward Primer Sequence** | **Reverse Primer Sequence** |
| --- | --- | --- |
| ESR1 | GTGCCTGGCTAGAGATCCTG | GGTTCCTGTCCAAGAGCAAG |
| TNF | GCCCATGTTGTAGCAAACCC | TATCTCTCAGCTCCACGCCA |
| SYNE1 | GGTCCCGGTATAAAGGCTCG | AGCCGCCCTCCTGGAGAT |
| PGR | TGGGCATTGTGGTCGGTATG | GAACAAGGAGCCAAGCGGTA |

**Additional File 3. Primer sequences for qPCR assay.**
